# The elaboration of exploratory play

**DOI:** 10.1098/rstb.2019.0503

**Published:** 2020-06-01

**Authors:** Maddie Pelz, Celeste Kidd

**Affiliations:** 1 Brain and Cognitive Sciences, Massachusetts Institute of Technology, 43 Vassar Street, Cambridge, MA 02139, USA; 2 Psychology, University of California, Berkeley, 2121 Berkeley Way, Berkeley, CA 94720, USA

**Keywords:** exploration, play, play complexity, touchscreens, cognitive development, developmental experimentation

## Abstract

We apply a new quantitative method for investigating how children's exploration changes across age in order to gain insight into how exploration unfolds over the course of a human life from a life-history perspective. In this study, different facets of exploratory play were quantified using a novel touchscreen environment across a large sample and wide age range of children in the USA (*n* = 105, ages = 1 year and 10 months to 12 years and 2 months). In contrast with previous theories that have suggested humans transition from more exploratory to less throughout maturation, we see children transition from less broadly exploratory as toddlers to more efficient and broad as adolescents. Our data cast doubt on the picture of human life history as involving a linear transition from more curious in early childhood to less curious with age. Instead, exploration appears to become more elaborate throughout human childhood.

This article is part of the theme issue ‘Life history and learning: how childhood, caregiving and old age shape cognition and culture in humans and other animals’.

## 1. Introduction

Exploration is a behaviour that agents employ to reduce uncertainty about their environments (e.g. [1–5]). The act of exploration trades new information in exchange for certain costs. First, there is the opportunity cost owing to the limited nature of space and time. For example, a monkey who decides to search previously unexplored foliage for fruit is necessarily missing out on the opportunity to exploit known food sources (i.e. the ‘explore/exploit dilemma’). Second, given that dangers exist in the world, there is a risk associated with novelty. Exploring new terrain or trying new things comes with new, unknown risks.

Despite these costs, animals broadly exhibit an array of curiosity-driven behaviours, including play, exploration, reinforcement learning, latent learning and neophilia (e.g. [6–8]). Empirical work has documented exploratory behaviour extensively in mammals [9–12], and also birds [13], crabs [14], bees [15,16], ants [17], moths [18] and even the humble roundworm *Caenorhabditis elegans* ([19]; see [20] for review).

Human curiosity, however, is singular. Humans explore more, and are willing to pay more for the opportunity, than other species [1]. The strength of this drive is apparent even in infancy [21–25]. By contrast, other primates exhibit *far less* curiosity and *far greater* neophobia in the wild (e.g. lemurs as in [26]), including those with comparatively long life histories like orangutans [27]. Even under ideal circumstances, in which the normal risks associated with exploration are virtually eliminated from the context, most primate species do not engage in anywhere near human rates of neophilia, independent exploration or innovation [11,28,29]. While we know that human levels of curiosity make our species unique, we still do not understand how this distinctively human attribute impacts the life history of the species. We suspect, however, understanding this facet of human life history is a part of the key to understanding why humans are such outliers in terms of both our intelligence, but also our rich cultural heritage [30].

The hyper-curiosity of humans, coupled with our protracted life history, has been popularly suggested to benefit members of the species by creating extra opportunities for both new innovation and the cross-generational transmission of cultural information, thereby saving individuals the time and energy that would be required to discover every innovation they employ to their advantage by themselves from scratch [31,32]. Further, the protracted life history of humans could serve to balance the delicate trade-off between exploring and exploiting by allowing members of the species to both explore broadly during childhood, while under the protective care of adults, and exploit after maturity, with the benefit of updated environmental information courtesy of a long, protected exploratory phase preceding the exploitation phase [33,34]. Data in line with this theory include those which show children entertain unusual hypotheses more readily than adults [34]. This theory represents one reasonable strategy for exploring the vast space of all possible information sources available in the world: an agent might explore broadly before focusing (e.g. [35,36]).

However, other contradictory theories have suggested that young children's behaviour does not resemble the predictions of rational explore/exploit theories. For example, Piaget and many other developmental psychologists have noted that young children engage in repetitive, perseverative play (e.g. [37]). Past research on exploration during play has largely favoured descriptive measures over controlled experimental designs (e.g. see [38] for a review). Further, past attempts to quantify exploration typically employed hand-coding specific exploratory behaviours for interacting with objects, thus converting inherently rich and complex interactions to a comparatively coarse, somewhat arbitrary set of behaviours before analysis. For example, Caruso [2] recorded each time a child performed actions such as ‘squeeze’, ‘shake’, ‘bang’ or ‘twist’ as she explored a novel object. These coarse, time-consuming measures made it difficult for researchers to expand beyond assessing the way in which a child explores only one novel object that has been separated from any causal structure or relationship to its environment. Coarse quantitative metrics also limited what researchers could understand about how exploration might change with age, or what purpose any changes might serve under a rational framework.

Several recent studies have also taken quantitative approaches to studying exploration. These studies have focused on contexts in which the agent had a simple, specific learning task—for example, to discover a correct answer in a question-asking task [39,40], a correct label of items in a categorization task [41] or a choice that offers the highest reward in a *k-*arm bandit task [42]. These studies help to elucidate how school-age children narrow down and weigh evidence in order to arrive at a correct answer. They are, however, limited in how much they can reveal about exploration in unconstrained contexts, such as during free play. They are also uninformative about age-related changes in exploration strategies, as well as how developmentally related changes in resources, priorities and pressures impact behaviour. The majority of children's learning (about language, objects, categories and social structures in the world) occurs through free rather than forced exploration; thus, it is important to study exploration in less constrained contexts such as free play.

Here, we present, to our knowledge, the first quantitative assessment of changes in exploration across childhood, from toddlerhood to adolescence, in order to investigate age-related changes in exploration strategies. Our work quantitatively charts how exploration develops across distinctly human development. Thus, it forms the crucial foundation necessary for future comparisons to other species. This work aims to understand how distinctive features of human life history may relate to exploratory pressures in learning in general. To do this, we introduce an information-theoretic quantification of the breadth and complexity of a child's play behaviour using a compression scheme in order to see how concisely their sequence of chosen exploratory actions can be described. Before we present our new empirical data, we begin by outlining what previous work tells us about curiosity and exploration in human childhood.

### (a) Uncertainty prompts exploration

Previous work has shown that children explore in order to understand the properties of objects themselves, as well as how they relate to or interact with other animate or inanimate entities in the environment. In contrast with Piaget's ‘trial and error’ description of exploratory play [37], we now know that children can and do explore in order to engage in hypothesis testing about causal structures [35,43–46]. In this way, exploration leads to a deeper understanding of a child's current surroundings as well as the construction of object categories used to predict the properties that might govern future objects or environments [47,48]. Exploration allows learners to go beyond simple observation in order to discover causal relationships and latent intermediate variables through direct intervention [38,47,49–51]. Exploration is selective, flexible and is driven by the goal of disambiguating the causal structure of the environment.

Uncertainty motivates exploration [52]. Learners preferentially explore things that are more highly confusable or go against previously held beliefs [53]. For example, children selectively explore objects that go against their explanations of the laws of balance, and explore objects that violate different laws of physics (e.g. defying gravity versus passing through a solid object) in different ways [38,54].

### (b) Developmental changes in exploration are poorly understood

It is likely that children explore in different ways across development. Recent work suggests that children explore broadly across a wide range of potential hypotheses (e.g. [35,35]), while older observational studies suggest children's exploration to be less broad and more repetitive in nature [37]. We know of no previous work aiming to reconcile these two seemingly contradictory theoretical accounts. However, understanding how these dynamics change across development is an important step to revealing more about the impact of exploratory behaviour on learning outcomes and the function of different phases in human life history.

### (c) Approach

Here, we aim to quantify the *complexity* and *efficiency* of exploration across development in a rich, flexible environment that engages children of vastly different ages. Children learn more when they are able to interact with and test their own hypotheses within an environment rather than simply observing demonstrations of causal relationships [55]. Touchscreens allow for young children to confidently control on-screen objects, and thus they enable more intuitive play than is possible with a traditional mouse or physical objects (which are difficult for young children to manipulate owing to underdeveloped fine-motor abilities).

In this study, we assessed exploration in the context of the touchscreen app called Toca Kitchen Monsters, developed by Stockholm-based Toca Boca. To collect these data, we used a custom-made build constructed to allow us to collect detailed action data on our devices in the laboratory. The digital environment of Toca Kitchen Monsters offers children many different possible objects and actions to explore, in an environment that the designers at Toca Boca intentionally created to emulate physical-world freeplay opportunities. The basic structure of the digital environment is that there are different monsters (two: brown or blue) who have different food preferences that children can discover. The child can select from a range of food ingredient options (eight: mushroom, tomato, broccoli, lemon, carrot, sausage, steak or monsterfood), preparation methods (six: chopping, blending, boiling, frying, microwaving or raw) and spicing options (three: salt, pepper or nothing) in order to prepare meals to feed to the monsters. A meal consists of only one food item, but it can be prepared using multiple preparation methods and a range of preparation and spicing options (e.g. lots of salt, a little salt and pepper, a little pepper only, none of either, chopped and boiled, boiled and blended, blended a lot or very little, etc.). Also, multiple meals can be prepared and set on a plate. They can be served one at a time, with preparation of each new meal between ‘feedings’, or in a row, for a kind of serial feasting-style feeding. Meals can also be prepared and never fed to the monster at all, just as a child could make a pretend meal with toys or physical objects and opt to not serve it to a friend or caretaker. If the food is offered to the monster, the monsters express a range of deterministic reactions to the meals (five: love, like, dislike, reject and spitting out), depending upon the combination of ingredients and preparation methods. For example, the brown monster expresses dislike for raw lemons, love for blended lemons and disgust for highly salted blended lemons. There are no explicit points awarded for finding new objects or cooking methods, no assigned goals, and no link between how elaborate the food is and the monsters' reactions. Players can determine their own subgoals within free play, which can be as simple as just taking foods out of the refrigerator and tossing them around to play with the relatively lifelike physics engine. Here, we investigate the complexity and efficiency of exploration by quantifying the engagement with different objects and actions within the digital environment, using the metrics detailed in table 1. These metrics quantify not just how many objects and actions are explored, but how efficiently they are discovered, and how much variety exists in exploration pattern sequences for each child.

**Table 1.** Measures.

variable	calculation
touch rate	total number of touches/length of play (300 s)
ratio of static touches	number of touches to inactive screen locations/number of total touches
discoveries per touch	number of unique objects found/total number of touches
play complexity	compressed size of file containing actions

In addition to their ease of use, touchscreens also allow for the collection of easily quantified data on exploratory behaviour in order to test theories about how exploration changes across development.

## 2. Methods

### (a) Participants

We tested a total of 132 children (ages 1 year and 10 months to 12 years and 2 months, mean (*M*) = 56.2, 45% female) who participated in the study in Rochester, NY, at the Rochester Baby Laboratory. We recruited them via mailings and in-person recruitment at community events. The participants were 81% White, 6% African American, 5% Asian, 4% other and 4% did not report. Our sample came from highly educated homes according to self-reports of the highest maternal educational achievements: 3% obtained a high school diploma, 2% completed less than 2 years of college, 12% obtained an associate's degree, 23% obtained a bachelor's degree, 37% obtained Master's degree, 3% obtained a Doctoral degree and 20% declined to respond. The children were all healthy and had normal or corrected-to-normal vision, and had no reported vision or hearing loss, according to parental report. Participating families received a small gift for the child and $10 to the parents for travel reimbursement.

### (b) Exclusion criteria

We excluded children if they did not play until the criterion time of 5 min (*n* = 21),^1^ had played Toca Kitchen Monsters before (*n* = 3) or whose parents were present during testing (*n* = 3). The final sample was 105 children (ages 22–146 months, *M* = 62.0). The food-only repetitive play analysis additionally excluded four children because they did not interact with any food items during their session (*n* = 101, 22–146 months, *M* = 62.7). Two additional children were not included in the touchscreen experience analysis because their caretakers did not complete the questionnaire, leaving that sample with an *n* = 103, 22–146 months, *M* = 62.4.

### (c) Materials

All participants played Toca Kitchen Monsters on a first-generation iPad Mini with the home button locked in order to prevent participants from exiting the app. We used a modified version of Toca Kitchen Monsters that saved touch action data (time, location, duration and object name) directly onto our tablets. We note that the publicly available Web-based version of this app does not store any touch data from users.

**Figure 1.**
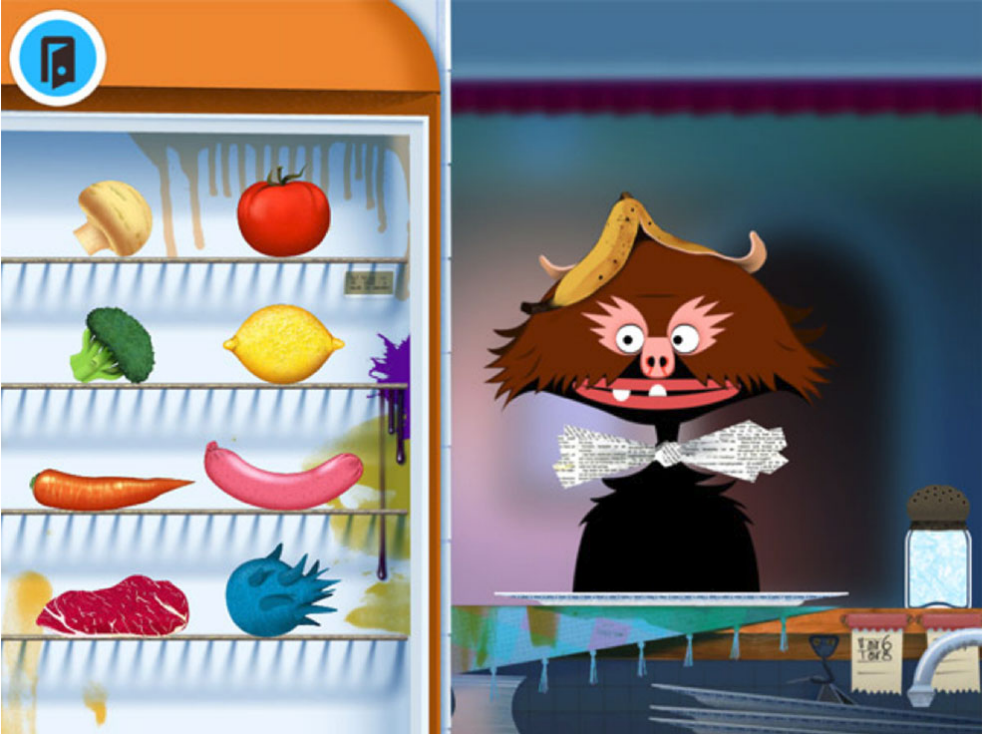
Screenshot from Toca Kitchen Monsters of the food item selection screen, which follows the monster selection screen.

The app consists of two monster characters, a refrigerator containing eight food items (mushroom, tomato, broccoli, lemon, carrot, sausage, steak and monsterfood) and a kitchen containing five appliances (a knife and cutting board for chopping, a blender for blending, a pot for boiling, a pan for frying and a microwave for microwaving), as well as salt and pepper. The food can be chosen and prepared using the appliances, and then fed to the monster in order to get feedback on his or her likes and dislikes, as described above. Figure 1 shows an example of the screen during play.

### (d) Procedure

**Figure 2.**
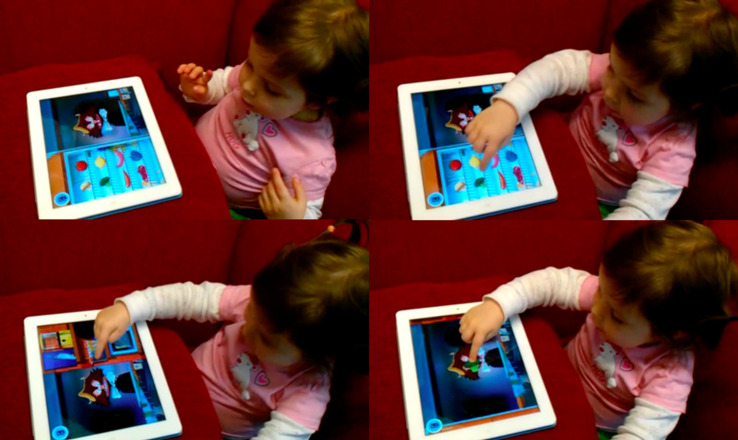
Two-year-old demonstrating play by considering food options in the refrigerator pane (upper left), selecting the broccoli by clicking and dragging (upper right), considering food preparation methods (lower left) and feeding the food to the monster by dragging it to his mouth (lower right).

Children played with toys in the waiting room of the laboratory in order to become comfortable with the researcher in a new environment before the experiment began. After the parent was briefed and consented to the study in the waiting room, a researcher accompanied the child to a testing room adjoining the main space. After the child was seated, the researcher demonstrated one touch on the tablet and then handed it to the child so that she could play freely (figure 2). Other than demonstrating the first touch, the researcher did not provide any instruction or guidance. The researcher also did not touch the tablet unless the participant minimized the app with an accidental touchscreen gesture. Play continued as long as the child was interested, for *a maximum of 10* *min*. After the session, the researcher accompanied the participant back to the waiting room.

### (e) Data analysis

Data consisting of the location, timing, target object and duration of each touch were automatically recorded and saved directly onto the tablet. Analyses were performed on the first 5 min (300 s) of data collected from each participant. This cut-off was established *a priori*, based on piloting work with an independent group of children of the same age range as those included in this sample. Most participants reached this threshold.

#### (i) Play complexity

In order to assess the complexity of each child's play, we developed a method for computing how much redundancy a child's play patterns contained by *computing the compressibility* of the sequential list of the actions they selected during play, controlling for length of the sequence. This method is akin to inferring that one digital text file contains more lexical and phrase-level repetitions than another of the same length because it reduces down to a smaller file size when it is converted to a ZIP archive file format. Higher values indicate both broader and more complex patterns of exploration.

Before compression, the names of the objects chosen were replaced with binary codes so that the string referencing each object was of the same length. The python gzip package was then used to output a compressed file using Lempel–Ziv compression. Behavioural sequences that compress to a higher degree suggest that behaviour was more repetitive, while those that are less compressed suggest stronger exploratory behaviour. Importantly, the compression measure incorporates the fact that ‘repetitive play’ may be non-trivial. For instance, a child who selects objects A, B and C in sequence ABCABCABC…. will appear maximally random to a model that tracks only unigram statistics, as A, B and C are equally frequent. This sequence will be very predictable to a compression scheme, like the one we use, that can capture inter-action dependencies. If past observations of perseveration in younger children ring true in our dataset, we would expect to see children to become less ‘sticky’ as they mature [37]. Total touches (as reflected in the length of the list to be compressed) were regressed out during analysis. This was done because we want to characterize the redundancy in the pattern of exploration, not simply the rate at which children are capable of making selections on a screen. Touch rate was also computed and assessed independently.

#### (ii) Touchscreen experience

Experience with touchscreens was calculated using a parental-report survey collected at the time of the study. The surveys were used to calculate average minutes per day of touchscreen use across different contexts (home, school, travel, etc.). By using a rate measure rather than a cumulative measure of touchscreen experience, the value is not necessarily confounded with age.

Four linear mixed-effects models were run to assess each of the measures described in table 1: one with age as the predictor, one with touchscreen experience as the predictor, one in which both age and experience were included as independent predictors and one that includes the interaction between age and experience as a predictor in addition to both age and experience as main effects.

## 3. Results

Results from comparing the four potential models using Akaike information criterion appear in the electronic supplementary material, appendix SII, and we report the results from the best-performing model below. Socioeconomic status as measured by maternal education was not a significant predictor of any of the following measures of touchscreen interaction and exploration.

### (a) Touchscreen interaction metrics

#### (i) Touch rate

**Figure 3.**
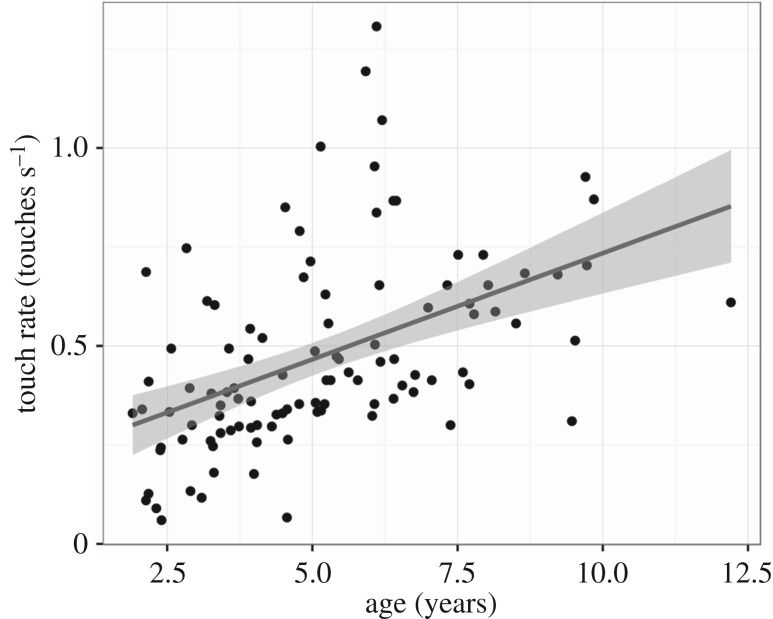
Touch rate increases with age (*n* = 105, *t* = 5.511, *p* < 0.0001).

*Predicting touch rate from age* (figure 3). The rate of touchscreen interactions increased significantly with children's age (*β* = 0.054, *t* = 5.511, *R*^2^ = 0.228, *p* < 0.0001).

### (b) Static touches

**Figure 4.**
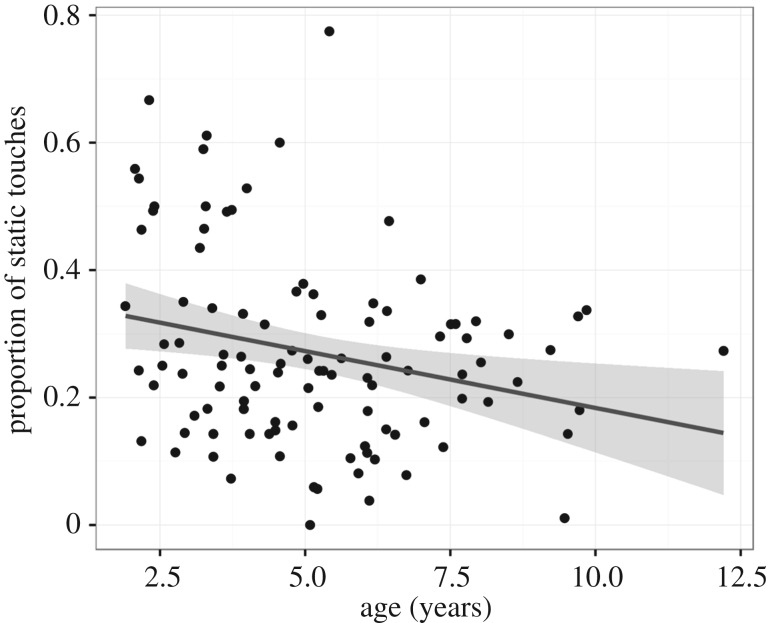
Proportion of static touches decreases with age (*n* = 105, *t* = −2.679, *p* = 0.009).

*Predicting proportion of static touches from age* (figure 4). The number of touches to inactive screen locations (static touches) decreases with age, with older children making more successful actions than younger children (*β* = −0.018, *t* = −2.679, *R*^2^ = 0.065, *p* = 0.009).

### (c) Efficiency of search metrics

#### (i) Discoveries per touch

**Figure 5.**
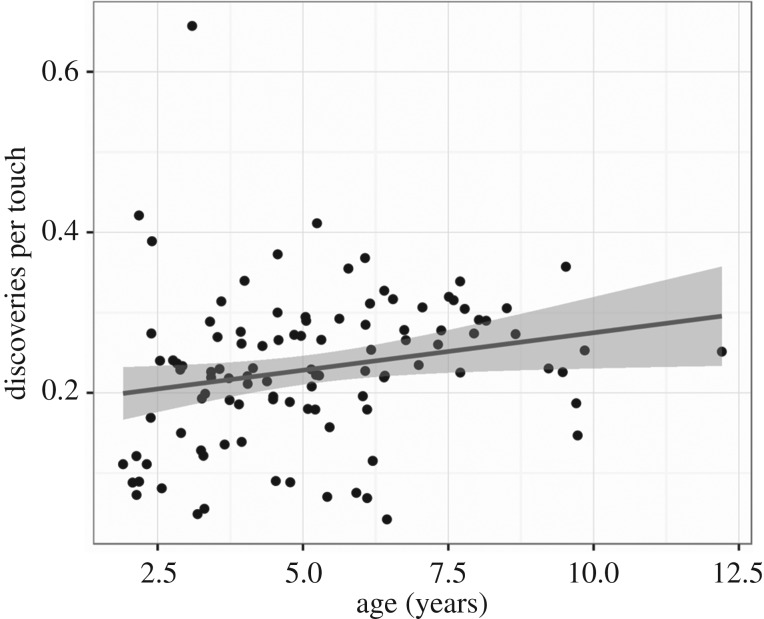
Discoveries per touch increase with age (*n* = 105, *t* = 2.20, *p* = 0.03).

*Predicting discoveries per touch from age* (figure 5). The number of novel items discovered per touch was calculated as a way to measure the efficiency of each participant's exploration of the touchscreen environment. This metric increases with age, suggesting that older children explore the breadth of options in the environment more efficiently (*β* = 0.0093, *t* = 2.20, *R*^2^ = 0.045, *p* = 0.03).

#### (ii) Play complexity

**Figure 6.**
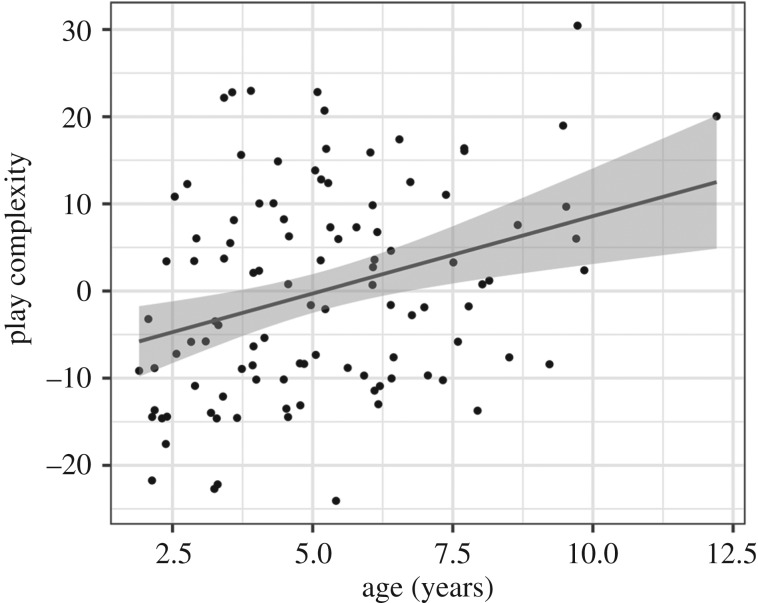
Play complexity (compressed file size residualized by total number of touches) increases with age in a model including age and touchscreen experience, suggesting that repetitive play decreases (*n* = 101, *t* = 3.92, *p* = 0.0002); this analysis used only the food items (omitting food preparation methods) in the interest of being conservative, because younger children may not have been able to navigate to the food preparation screen as readily. Conducting the analysis instead over all items yields similar (though strengthened) results (*t* = 5.68, *p* < 0.0001).

*Predicting play complexity from age* (figure 6). We measured play complexity (breadth and non-repetitive play patterns) using the previously described compression algorithm. Total touches (as reflected in the length of the list to be compressed) were regressed out, as we wish to characterize the redundancy in the pattern of exploration, not simply the rate at which children are capable of making selections on a screen. The best-fitting model predicting the degree of play complexity (compressed file size of touches) to the eight food objects includes the number of total touches, the child's age, and touchscreen experience (*R*^2^ = 0.169). Repetitive play decreased with age, with younger children showing more perseverative behaviours (*β* = 2.31, *t* = 3.92, *p* = 0.0002). Experience was not a significant predictor (*β* = 0.03, *t* = 1.37, *p* = 0.175). These findings are also robust for analyses of all objects in the environment beyond just the food items (*R*^2^ = 0.670), with play complexity increasing with age (*β* = 11.82, *t* = 5.68, *p* < 0.0001) and experience remaining non-significant (*β* = 0.01, *t* = 0.12, *p* = 0.9) (see the electronic supplementary material, appendix SI).

## 4. Discussion

### (a) Elaboration of exploratory play with age

Analysis of children's exploration in the modified Toca Kitchen Monsters environment allowed us to quantitatively examine changes in exploration across age. Toca Kitchen Monsters gave us a rich environment that was engaging to a wide age range, while controlling for differences in task structure that may have led to seemingly contradictory observations in the past. Older children completed more successful actions on the touchscreen, discovered more unique objects in the environment, showed increased efficiency of exploration and less repetitive play behaviour. In other words, children's play became more elaborate and efficient with maturation. This evidence suggests that children become more broadly exploratory over time, in line with older theories of exploration such as those espoused by Piaget [37].

In the current study, older children explored more widely, while younger children repeated their actions and limited the scope of exploration to a smaller subset of what was available to explore. Increasing play complexity (enabled by decreasing repetitive play) could allow the child to create new food preparations or combinations that would otherwise be left undiscovered.

### (b) Ecological validity and implications of task choice

We chose our task because it enabled us to understand children's default mode of exploration, by which we mean exploration in a context devoid of explicit goals and rewards (e.g. ‘points’). This is important in a life-history context because unstructured time is a hallmark of human childhood [56]. Our results suggest that, in the absence of explicit goals or rewards, children explore more broadly and in more complex patterns as they age, at least up until 12 years of age. While the digital toy environment cannot replicate some aspects of physical play (e.g. rotating objects to view them at all angles, as is common practice for young children [57]), it makes other exploratory actions possible. For example, younger children's exploration is hindered by their limited fine-motor abilities in real-world play. This prerequisite to physical exploration is greatly reduced for touchscreen play, because all that is required is a touch-and-drag action.

The use of tablets also offered us numerous methodological benefits. The greatest benefit was the ability to offer children many varied objects to explore and ways to explore them, free from the practical coding considerations that would restrict the set-up to a limited pool of offered options. Our task was engaging, as evidenced by the relatively long playtimes across all children of all ages who completed the task. For contrast, while we used a 5 min minimum play criterion window (and most children played for far longer), most typical physical play exploration tasks are able to elicit only a few minutes of free play (e.g. 0–2 min in [58–60]). This higher degree of engagement was made possible by the richness of the play environment and would have been difficult to achieve with a physical toy set-up that allowed coding to remain feasible.

### (c) Exploration across the lifespan

This study showed a stable trend towards elaboration of exploration during free play for young children through early adolescence. But we do not yet know how exploration might change from adolescence through to adulthood. Existing work on curiosity across the lifespan generally tends to focus on the stability of curiosity as a trait, and overwhelmingly employs survey-based methods over actual observations of exploratory behaviour. Previous studies typically report modest declines in subjective feelings of curiosity with increased age in adults (e.g. [61–63]), but these declines are often limited to certain aspects of curiosity (e.g. interpersonal curiosity) and do not always apply across the board (e.g. [61]; the trend is driven only by women reporting interpersonal curiosity and, even then, they note that the change is ‘modest’). The trait literature suggests that ‘openness to new experiences' may decline with age (e.g. [64–66]). If these data were to indicate that curiosity declined with age, it could suggest that we should expect to see an inverted-U-shaped trend in exploration across the lifespan. However, we do not have nearly enough evidence to conclude any such thing at this point. Subjective reports of curiosity are not the same thing as actual exploration in the world**.** What we would ideally want is access to something like Internet search histories for individuals across their lifespan, presuming the ethics of gathering such a dataset were well managed. We are not aware of any dataset at this time appropriate for comparing across ages for the entire lifespan.

Under a rational life-history framework, we should understand that the precise way in which an agent *should* explore should change as their abilities, needs and priorities change across their lifespan; and changes in abilities, needs and priorities are inextricably linked to environmental factors (e.g. [67]). For example, food scarcity leads to lower levels of parental care provided by adult mice [68], and mice pups leave the nest earlier and go through puberty sooner in response to lower levels of parental care [69,70]. Young mice also undergo puberty sooner in these conditions [71]. These impacts are consistent with the *stress acceleration hypothesis* (e.g. [72]), by which environmental hardships trigger young offspring to ‘gear up’ early in ontogeny for an existence of fewer resources and less support. Under these circumstances, these offspring have shorter protected periods for exploration available to them. Thus, it would be an oversimplification to expect for general trends in changes to change only with age, as opposed to a complex interaction of a number of different factors and pressures across the lifespan. Further work on this area would promote a better understanding of age-related changes in exploratory behaviour from an evolutionary and life-history perspective, though there remain some practical and technical challenges in collecting such a dataset.

### (d) Safe environments enable neophillia

An important caveat of this work is that the touchscreen environment we employed allowed children to explore in context with no real penalties or real-world risks. While our intention was to explore age-related changes in exploration patterns and the situation was kept constant in this way across the ages, future work will examine the degree to which our results generalize to real-world environments in which safety is not guaranteed.

### (e) What developmental change is responsible for this behavioural shift?

While the age-related elaboration of exploration is apparent in our data, identification of which of many possible developmental changes is most responsible for the observed behavioural shift requires further inference. Most likely, it is not one aspect of development that is entirely responsible for the observed shift, but multiple aspects and interactions. The possibilities can be described as falling into at least three categories: age-related changes in *core cognitive capabilities* (like working memory, response inhibition and attention), age-related changes in *goals or priorities*, and age-related *differences in learning*.

The first possibility is that increased efficiency in exploration hinges on changes in a child's *core capabilities*, such as working memory, which increases over the course of childhood (e.g. [73–75]). The increase in working memory could directly result in more efficient exploration. While older children could hold more information in their working memory and learn relationships between objects more quickly, younger children might rely on more repetitive evidence to establish and encode causality, leading to a less broad scope of exploration. Similarly, age-related increases in executive function could be required to enable children to disengage more readily from particular sets of actions.

A second possibility is that the increased efficiency stems from an age-related *shift in priorities*. For example, older children may be more motivated to explore for the purpose of understanding the causal relationships in the scene, or perhaps some shift in social motivation like finding options that please the monsters. While this is a theoretical possibility, we believe this to be less likely. Nothing in our data suggests that younger children are less interested in the causal structure of the set-up. If we did, we might expect that they would engage in feeding the monster less, perhaps favouring just exploring the food options and preparation methods themselves, which we do not. Further, and more convincingly, existing studies suggest that even very young children can disambiguate causal relationships and that their drive to do so motivates exploration (e.g. [38,47,50,55,58]). Likewise, even toddlers much younger than the children tested here exhibit the ability to represent what other agents want and a desire to accommodate them accordingly [76], suggesting that children in our age range would all be motivated to appease the monster and, perhaps to some degree, the experimenter who introduced the touchscreen toy.

A more likely possibility might be that working memory and the desire to discover causal structures interact to result in the observed age-related differences. For example, older children with increased working memory capacity are able to test multiple hypotheses at once using a discriminative strategy, while younger children are restricted to single hypothesis testing [77]. It is also possible that these differences in working memory and causal understanding limit younger children from understanding the overarching structure of the digital environment and cause them to play within a subset of options, while older children may be able to grasp the concept of the different food and preparation combinations available.

Finally, the third possibility is age-related *variation in learning*. Perhaps, younger children appear to explore less because of failures to learn the structure of the digital play environment. While this is a theoretical possibility, and we note that we do not directly test learning outcomes here, we do not think lack of learning is likely as an explanation because of the vast existing literature demonstrating that much younger children can learn large quantities of statistical associations between events in the world very quickly, even during passive paradigms (e.g. [78,79]).

### (f) Experiential differences

In contrast with its many benefits, incorporating touchscreens into study designs also carries a possible confound owing to children's previous touchscreen experience. Every participant in the current study reported some level of touchscreen experience, and although the measure we used was explicitly not cumulative, there is still the possibility that older children have built up experience over time. This leaves the possibility that some of the effects that we attributed to maturation might be influenced by compounded experience; thus, it would be interesting to replicate this experiment in a population with limited prior access to touchscreen technology.

### (g) Trait-level differences

It is worth noting that there is more variance within ages than one might expect, with especially the youngest age ranges showing a wide range of exploration efficiency (with only 14% of the overall variance in our exploratory complexity measure accounted for by age). The degree to which individual children would demonstrate variance in their exploratory behaviour across different contexts or whether exploratory efficiency is a fixed trait remains an interesting open question. In this task, we used a cohort design in order to measure changes in exploration across development, so additional follow-ups could include assessing individual children's exploratory behaviour over time to further explore the individual variance in exploration efficiency. Follow-up work should include longitudinal data and intervention studies to explore causality.

It is very possible that different exploratory behavioural patterns early in life could give rise to what would appear to be a trait-level difference. Cross-species work may be helpful in testing this idea. For example, Freund *et al*. [80,81] used a measurement of spatial exploration patterns that they called *roaming entropy*, and demonstrated that laboratory-bred genetically identical mice developed increasingly different levels of spatial exploration with experience. The same might be true of humans' exploration, but further work would be required to determine whether this was in fact the case.

## 5. Conclusion

We observe that children become more broadly exploratory between their toddlerhood and adolescence, as assessed in a digital play environment. These findings cast doubt on the picture of human life history as involving a linear transition from highly exploratory in infancy to more specialized in adulthood as a solution for solving the tensions between exploring the environment and exploiting known resources. Instead, the changes we see throughout childhood suggest that if such a tendency exists, it is part of a more complex system that interacts with other aspects of development, such as age-related changes in working memory and response inhibition.

## Supplementary Material

Appendix I & II

## Endnote

1 Seven of these children were under the age of 22 months—none of the participants under that age made it to criterion, so no further children in that age group were tested. This lower age bound is probably owing to the design and goals of this specific app rather than the limitations of the touchscreen device itself.

## Data accessibility

The data and analysis files may be accessed via the senior author's github account at https://github.com/celestekidd/.
