# Supplementary material for: The elaboration of exploratory play
Source: Philos Trans R Soc Lond B Biol Sci. 2020 Jun 1;375(1803):20190503. doi: 10.1098/rstb.2019.0503 (PMC7293158; doi:10.1098/rstb.2019.0503)
Supplement: Appendix I & II [file rstb20190503supp1.pdf]

The Elaboration of Exploratory Play

Supplementary Materials

Maddie Pelz[*,☆] & Celeste Kidd[+,☆]

* Brain & Cognitive Sciences, Massachusetts Institute of Technology, 43 Vassar St, Cambridge, MA 02139
+ Psychology, University of California, Berkeley, 2121 Berkeley Way, Berkeley, CA 94720
☆ Corresponding authors: mpelz@mit.edu, celestekidd@gmail.com

Appendix I - Table of Results

A. **Touch Rate**

Predictor	β	Std. Error	t-value	p-value
Age	0.054	0.010	5.51	2.65e-07

B. **Static Ratio**

Predictor	β	Std. Error	t-value	p-value
Age	-0.018	0.007	-2.68	0.009

C. **Discoveries Per Touch**

Predictor	β	Std. Error	t-value	p-value
Age	0.009	0.004	2.20	0.03

D. **Play Complexity (Compressed File Size for Food Only Analysis, Residualized by Total Touches)**

Predictor	β	Std. Error	t-value	p-value
Age	2.310	0.590	3.92	0.000165
Experience	0.030	0.023	1.367	0.175

E. **Play Complexity (Compressed File Size for All Item Analysis, Residualized by Total Touches)**

Predictor	β	Std. Error	t-value	p-value
Age	11.810	2.080	5.68	1.37e-07
Experience	0.010	0.082	0.119	0.905

Appendix II - Model comparison

Model	AIC	R^2
TouchRateByAge	-23.091	0.228
TouchRateByExp	1.876	0.030
TouchRateByAgeAndExperience	-20.444	0.234
TouchRateInteraction	-18.473	0.235
StaticTouchByAge	-102.814	0.065
StaticTouchByExperience	-95.407	0.021
StaticTouchByAgeAndExperience	-100.547	0.087
StaticTouchInteraction	-99.757	0.098
DiscPerTouchByAge	-197.269	0.045
DiscPerTouchByExp	-188.272	0.006
DiscPerTouchByAgeAndExperience	-190.569	0.047
DiscPerTouchInteraction	-189.227	0.053
ComplexityByAgeAndTotalTouches (only food objects)	812.162	0.144
ComplexityByExperienceAndTotalTouches (only food objects)	809.555	0.040
ComplexityByAgeAndExperienceandTotalTouches (only food objects)	796.720	0.169
ComplexityInteraction (only food objects)	798.717	0.169
ComplexityByAgeAndTotalTouches (all objects)	1074.523	0.670
ComplexityByExperienceAndTotalTouches (all objects)	1083.814	0.562
ComplexityByAgeAndExperienceandTotalTouches (all objects)	1056.783	0.670
ComplexityInteraction (all objects)	1058.400	0.671
